# Supplementary material for: The interactome of intact mitochondria by cross-linking mass spectrometry provides evidence for coexisting respiratory supercomplexes
Source: Mol Cell Proteomics. 2017 Dec 8;17(2):216–32. doi: 10.1074/mcp.RA117.000470 (PMC5795388; doi:10.1074/mcp.RA117.000470)
Supplement: Supplemental Data [file supp_RA117.000470_133922_0_supp_23978_fzffwf.zip › spectra_annotation/mito_spectra_annotation/349-1-6-1-9-1.pdf]

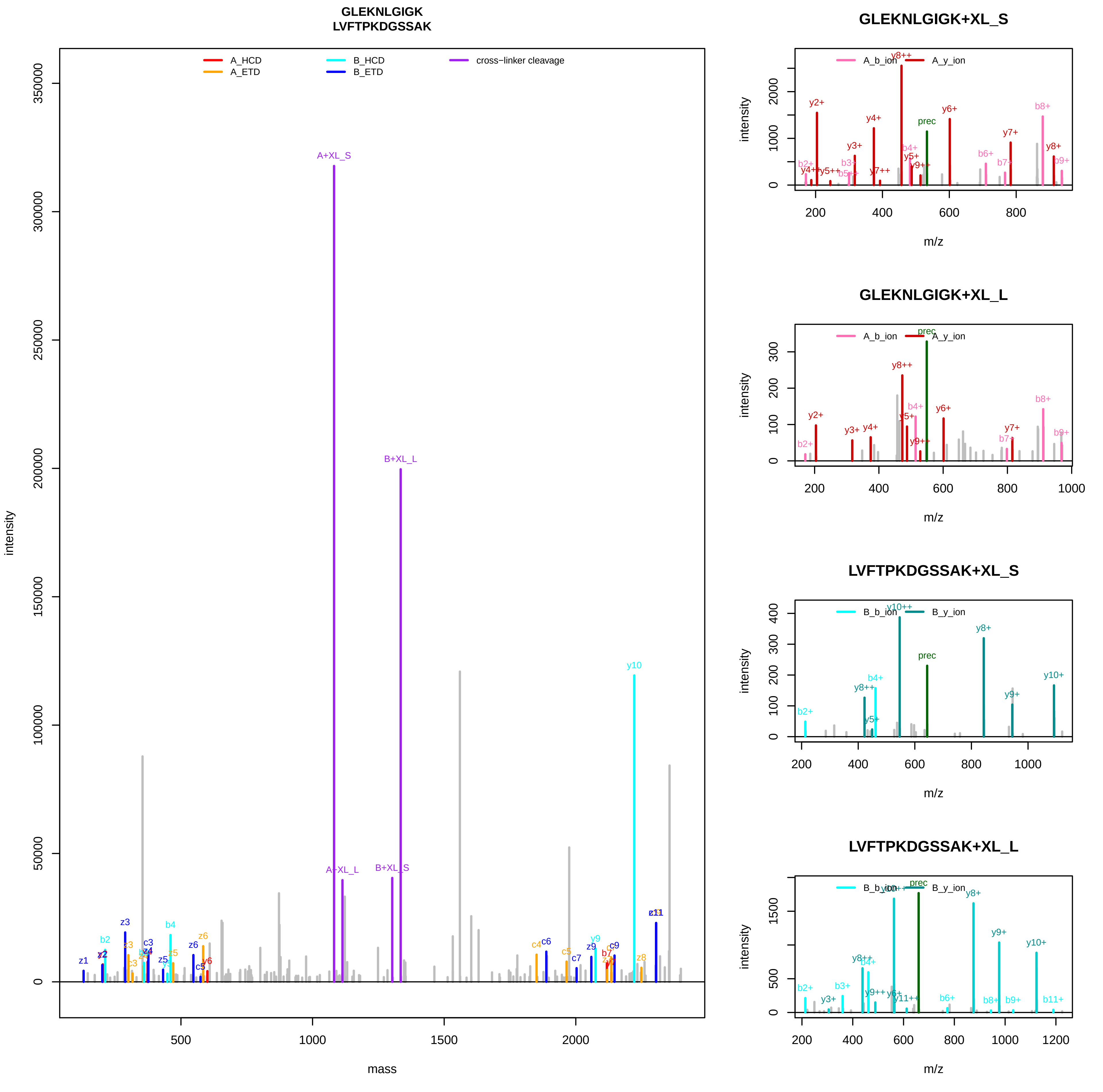

GLEKNLGIGK
LVFTPKDGSSAK
A_HCD
A_ETD
B_HCD
B_ETD
cross−linker cleavage
A+XL_S
B+XL_L
A+XL_L
B+XL_S
y10
intensity
mass
0
50000
100000
150000
200000
250000
300000
350000
500
1000
1500
2000
GLEKNLGIGK+XL_S
A_b_ion
A_y_ion
y8++
y2+
y4+
y3+
y6+
prec
b8+
y7+
y8+
b9+
b6+
b7+
intensity
m/z
0
1000
2000
200
400
600
800
GLEKNLGIGK+XL_L
A_b_ion
A_y_ion
prec
y8++
b4+
y6+
y2+
y3+
y4+
y7+
b8+
b9+
b2+
intensity
m/z
0
100
200
300
200
400
600
800
1000
LVFTPKDGSSAK+XL_S
B_b_ion
B_y_ion
y10++
y8+
prec
b4+
y8++
y10+
y9+
b2+
y5+
intensity
m/z
0
100
200
300
400
200
400
600
800
1000
LVFTPKDGSSAK+XL_L
B_b_ion
B_y_ion
prec
y8+
y9+
y10+
y8++
b4+
b2+
b3+
y3+
y9++
b6+
b8+
b9+
b11+
intensity
m/z
0
500
1500
200
400
600
800
1000
1200
